# Supplementary material for: A reliable in vitro rumen culture system and workflow for screening anti-methanogenic compounds
Source: PLoS One. 2025 Dec 1;20(12):e0335844. doi: 10.1371/journal.pone.0335844 (PMC12668615; doi:10.1371/journal.pone.0335844)
Supplement: S1 File — (PDF) [file pone.0335844.s001.pdf]

Oct 24, 2025

In vitro Cultivation and Microbiome Analysis

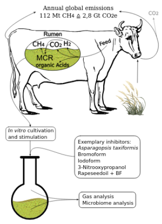

DOI
dx.doi.org/10.17504/protocols.io.kxygxwrnkv8j/v1

Philip Laric[1], Armina Mortazavi[1,2], Ewa Węgrzyn[3], Kathrin Simon[1], Pauline Sophie Rittel[4], Florain Markus Trefz[4], Benedikt Sabass[1,2,5]

[1]Department of veterinary science, LMU Munich, 81377, Germany;
[2]Faculty of Physics and Center for NanoScience, LMU Munich, 80752, Germany;
[3]Institute for Chemical Epigenetics (ICEM), LMU Munich, 81377, Germany;
[4]Clinic for Ruminants with Ambulatory and Herd Health Services, Center for Clinical Veterinary Medicine, LMU Munich, 85764 Oberschleißheim, Germany;
[5]Department of Physics, TU Dortmund University, 44221 Dortmund, Germany

Philip Laric
vetmed. department AG Sabass

Create & collaborate more with a free account

Edit and publish protocols, collaborate in communities, share insights through comments, and track progress with run records.

Create free account

OPEN ACCESS

DOI: https://dx.doi.org/10.17504/protocols.io.kxygxwrnkv8j/v1

Collection Citation: Philip Laric, Armina Mortazavi, Ewa Węgrzyn, Kathrin Simon, Pauline Sophie Rittel, Florain Markus Trefz, Benedikt Sabass 2025. In vitro Cultivation and Microbiome Analysis. **protocols.io**
https://dx.doi.org/10.17504/protocols.io.kxygxwrnkv8j/v1

License: This is an open access collection distributed under the terms of the **Creative Commons Attribution License**, which permits unrestricted use, distribution, and reproduction in any medium, provided the original author and source are credited

Protocol status: Working
We use this collection and it's working

Created: February 20, 2025

Last Modified: October 24, 2025

Collection Integer ID: 124080

Keywords: In vitro rumen cultivation, Methane inhibition, Microbiome analysis, PCR, Next generation sequencing, Greenhouse gas, ruminants, Cow, functioning rumen cultivation setup, rumen cultivation setup to screen, rumen cultivation, other rumen cultivation, ruminal culture, analysis of the microbiome, microbiome analysis, microbiome, ruminal, microbiome analysis this protocol, cultivation

Funders Acknowledgements:
German Federal Ministry of Education and Research (BMBF)
Grant ID: 031B1504
European Union's Horizon 2020 research and innovation programme
Grant ID: 852585

Abstract

This protocol describes the steps for building a setup for *in vitro* rumen cultivation. The full workflow of inoculation of ruminal cultures, measurement of produced gases, as well as the analysis of the microbiome is described. As result one should be able to build a fully functioning rumen cultivation setup to screen for compounds of interest or to conduct any other rumen cultivation related studies.

Materials

General equipment:

- 8-Channel pipette (Ergonomic High Performance multichannel pipette, VWR, Darmstadt, Germany)
- Autoclave (HG-133, HMC-Europe, Tüßling, Germany)
- Epoxy resin (Epoxyplast 100 P, DIPON.DE, Dortmund, Germany)
- Falcon tubes (Sarstedt, Nümbrecht, Germany)
- Glue gun (TC-GG 30, Einhell Germany, Landau/Isar, Germany)
- Luer-Lock adaptor female/female (neoLab, Heidelberg, Germany)
- Luer-Lock barbed adaptor 6mm on female and male (neoLab, Heidelberg, Germany)
- Luer-Lock valve male/female (Discofix®, B.Braun, Melsungen, Germany)
- Magnetic stirrer (ROTILABO®MH-15, Carl Roth, Karlsruhe, Germany)
- Pipettes 2 - 1000 µL (Eppendorf, Hamburg, Germany)
- Reaction tubes (Eppendorf, Hamburg, Germany)
- Serological pipettes (Sarstedt, Nümbrecht, Germany)
- Silicon tubes Ø 6mm (ESSKA, Hamburg, Germany)
- Vaseline (Heinrich Hagner GmbH, Freudenstadt, Germany)

Troubleshooting

Files

SEARCH

Protocol

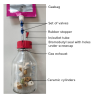

NAME

Setup design

VERSION 1

CREATED BY

Philip Laric
vetmed. department AG Sabass

OPEN →

Protocol

NAME

In vitro cultivation

VERSION 1

CREATED BY

Philip Laric
vetmed. department AG Sabass

OPEN →

Protocol

NAME

Gas measurements

VERSION 1

CREATED BY

Philip Laric
vetmed. department AG Sabass

OPEN →

Protocol

NAME

Microbiome analysis

VERSION 1

CREATED BY

Philip Laric
vetmed. department AG Sabass

OPEN →

Protocol

NAME

Data evaluation

VERSION 1

CREATED BY

Philip Laric
vetmed. department AG Sabass

OPEN →
