# Supplementary material for: A reliable in vitro rumen culture system and workflow for screening anti-methanogenic compounds
Source: PLoS One. 2025 Dec 1;20(12):e0335844. doi: 10.1371/journal.pone.0335844 (PMC12668615; doi:10.1371/journal.pone.0335844)
Supplement: S2 File — (PDF) [file pone.0335844.s002.pdf]

Oct 24, 2025

Setup design

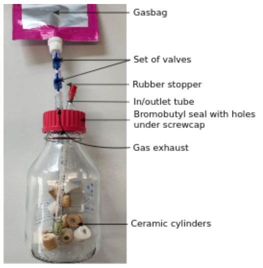

In 1 collection

DOI
dx.doi.org/10.17504/protocols.io.q26g7mq5qgwz/v1

Philip Laric[1]

[1]Department of veterinary science, LMU Munich, 81377, Germany

Philip Laric
vetmed. department AG Sabass

Create & collaborate more with a free account

Edit and publish protocols, collaborate in communities, share insights through comments, and track progress with run records.

Create free account

OPEN ACCESS

DOI: https://dx.doi.org/10.17504/protocols.io.q26g7mq5qgwz/v1

Protocol Citation: Philip Laric 2025. Setup design. **protocols.io** https://dx.doi.org/10.17504/protocols.io.q26g7mq5qgwz/v1

License: This is an open access protocol distributed under the terms of the **Creative Commons Attribution License**, which permits unrestricted use, distribution, and reproduction in any medium, provided the original author and source are credited

Protocol status: Working

Created: February 20, 2025

Last Modified: October 24, 2025

Protocol Integer ID: 124079

Keywords: In vitro, rumen, rumen simulation, anaerobic cultivation, custom laboratory system, setup design, setup design the setup design, design, epoxy resin, silicon tubing, using epoxy resin, controlled fluid handling, fluid handling, airtight seal, precise fluid management, setup, reliable seal, ideal for experiment, gas bag, component, experiment, lock valve, system

Abstract

The setup design focuses on creating a custom laboratory system by using epoxy resin to assemble components such as a screwcap, pipettes, and silicon tubing. The system is reinforced to ensure an airtight seal and includes a gas bag with a luer-lock valve for controlled fluid handling. The design ensures durability and a reliable seal, making it ideal for experiments that require secure and precise fluid management.

Materials

Reagents

- 3 D Printer (PRUSA i3 MK3, Prusa Research, Prag, Czech Republic)
- 500 mL GL45 laboratory bottles (DURAN® pressureplus, DWK Life Sciences, Mainz, Germany)
- 500 mL Spout bags (Daklapack, Lelystad, Netherlands)
- Bromobutyl plug seal closure (DURAN®, DWK Life Sciences, Mainz, Germany)
- Duct tape (3M, Neuss, Germany)
- Engraving tool (Dremel 3000, Dremel Europe, Breda, Netherlands)
- High-speed milling set (Dremel Europe, Breda, Netherlands)
- Open topped screw cap GL45 (DURAN®, DWK Life Sciences, Mainz, Germany)
- Polyethylene terephthalate filament (PET filament)(Polymaker, Houten, Netherlands)
- Toolbox (Conmetall Meister, Celle, Germany)

optional:

- Exsiccator (554, Kartell, Novoglio, Italy)
- Incubator (INCU-Line® IL56, VWR, Darmstadt, Germany)
- Resin colour or pigment (DIPON.DE, Dortmund, Germany)
- Vacuum pump (MZ 2 NT, Vacuubrand, Wertheim, Germany)
- Waterbath (WB-12, Phoenix Instrument, Garbsen, Germany)

Software

- Prusa Slicer (Prusa Research, Prag, Czech Republic)
- Solidworks 2017 (Dassault Systèmes, Stuttgart, Germany)

Evaluation

- UCSF ChimeraX v. 1.9 **[1]**
- GraphPadPRISM 5 (GraphPadSoftware, Boston, Massachusetts, USA)
- Inkscape 1.4 (Inkscape Community, Brooklyn, New York, USA)
- LibreOffice 24.8.4 (The Document Foundation, Berlin, Gemany)

Troubleshooting

Reagent setup: Setup design

1 **Epoxy resin:** mix two parts of component A with one part of component B. Stir thoroughly.

Note

Use alcohol ink or pigment to ensure proper mixing of the components. To shorten the pot life of the resin and enhance mixability, warm the components in a water bath.

Screwcap assembly

2

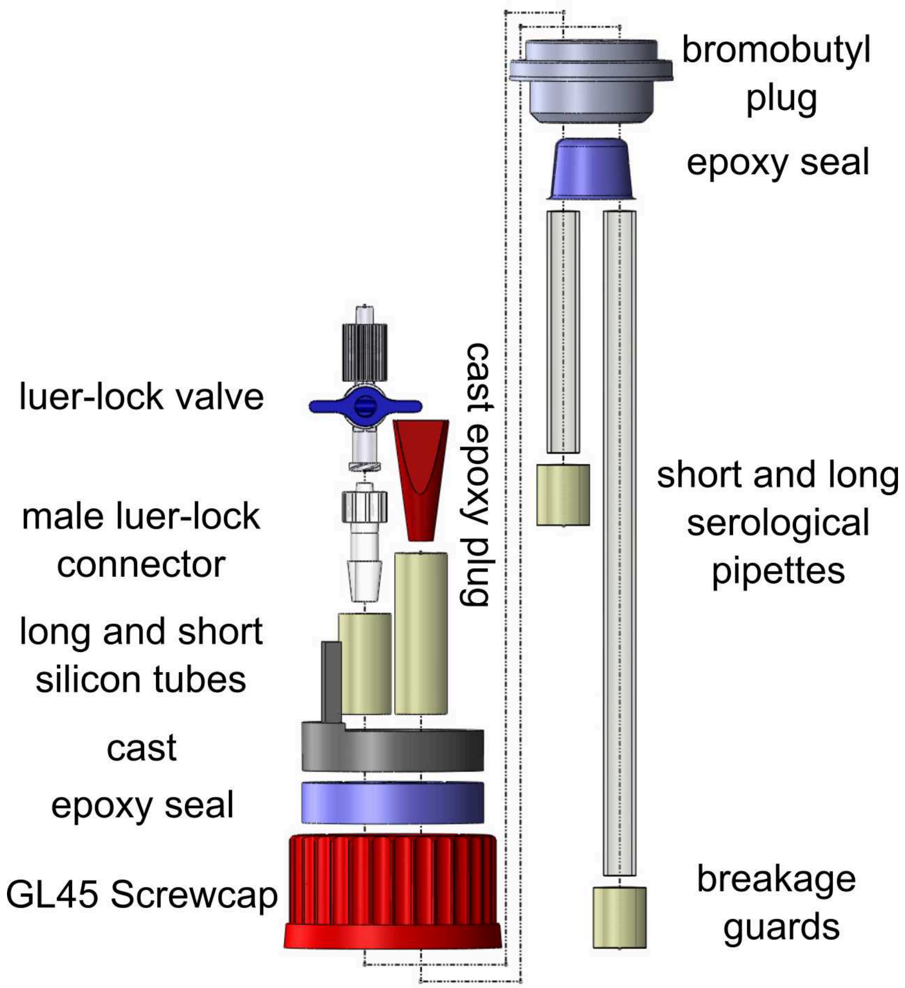

Exploded view of the screwcap assembly.

3

Note

The main vessel consists of a 500 mL GL45 pressure plus laboratory bottle.

To construct the plug, drill two holes, approximately 5 mm in diameter, into two opposing cavities of the bromobutyl stopper.

Note

CAUTION
Drilling is mostly done by friction, therefore hot flakes of rubber may fly around

4 Cut two 5 mL serological pipettes using a circular saw for the engraving tool to a length of 165 mm and 60 mm to yield a long and a short piece, respectively. Deburr the edges with a cutter knife.

5 Cut four pieces of silicone tubing to 1, 1, 2.5, and 4 cm lengths.

6 Insert the long pipette piece through the first hole, ensuring it reaches close to the bottom of the flask and protrudes 1 cm at the top.

7 The short piece should just penetrate the plug for about 2 cm and protrude 1 cm on top. Place a 1 cm long piece of silicone tubing over the lower ends as a breakage guard. The upper end gets attached to the 4 cm long silicone tube.

8 Ensure that the lower part of the bromobutyl stopper is not warped and that the pipettes stand straight, then cast it with epoxy.

9 Draw a cast on top of the screwcap with a diameter of the screwcap and a height of 1 cm. The reinforcement for the long silicone tube is optional.

10 Fix the mold with hot glue and seal all gaps. Cast the mold with epoxy and let it cure.

11 Insert the male luer-lock adaptor into the long silicon tube and secure the luer-lock valve on it

12 For the short silicone tube, either use a fitting hard rubber stopper, or 3D print a mold, cast it with epoxy and plug it in.

13 Screw the finished cap onto the glass bottle and check for leakage.

Note

Apply slight pressure to the bottle and submerge in water to check for the leakage.

Gasbag assembly

14

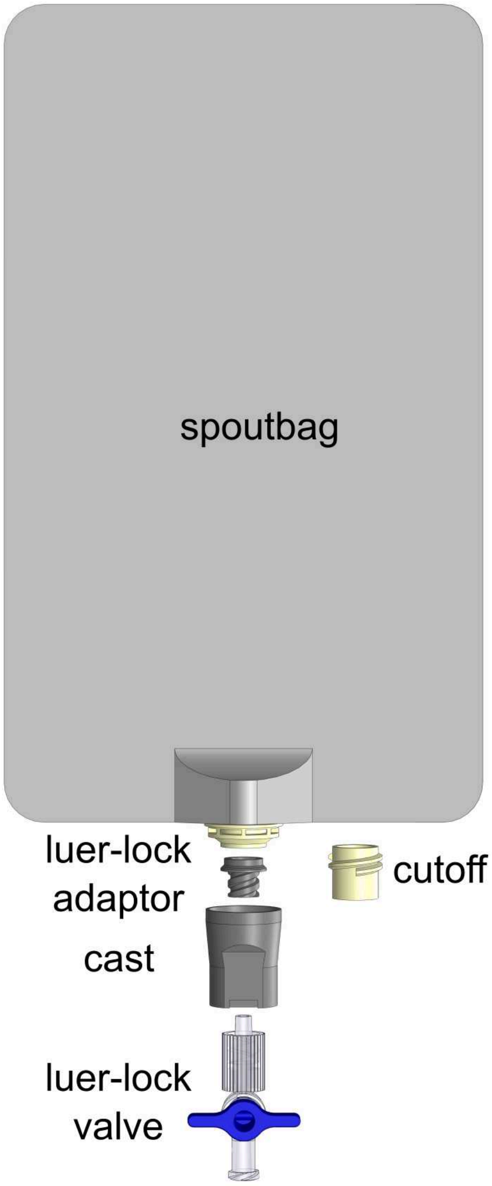

Exploded view of the gasbag assembly.

15 Take a spout bag and reinforce all edges and kinks with duct tape.

16 Cut the spout off.

17 3D print a female luer-lock adaptor that fits in the hole of the spout bag.

18 Attach a luer-lock valve and use hot glue to secure the adapter into place and seal it off.

19 3D print a cast around the spout and the luer-lock valve to allow easy manipulation of the valve.

20 Cast with epoxy.

21 Check for leakage.

Synthesis of 3-nitrooxypropanol

1d 1h

22 Add a solution of 3-bromo-1-propanol (2 g , 1.3 mL , 14.4 mmol , 1 eq) in acetonitrile (10 mL) to a solution of $AgNO_3$ (silver nitrate) (3.67 g , 21.6 mmol , 1.5 eq) in acetonitrile (20 mL) and stir at Room temperature for 24:00:00 .

1d

23 To the reaction mixture, add brine (150 mL) and stirred for 01:00:00 . Filter the silver salts through Celite and extract the filtrate with diethyl ether (3 × 100 mL). Wash the organic layer with brine, dry over disodium sulfate, filter, concentrate and dry under vacuum. The product is obtained as a yellow liquid (0.847 g , 6.99 mmol , 49% yield).

1h

24 Conduct the analysis of 3-nitrooxypropanol via ^{1}H and ^{13}C nuclear magnetic resonance (NMR), high-resolution mass spectrometry (HRMS) and infrared spectroscopy (IR).

Protocol references

[1] Pettersen EF, Goddard TD, Huang CC, Meng EC, Couch GS, Croll TI, et al. UCSF ChimeraX: Structure visualization for researchers, educators, and developers. Protein Sci Publ Protein Soc. 2021 Jan;30(1):70–82.
