# Supplementary material for: A reliable in vitro rumen culture system and workflow for screening anti-methanogenic compounds
Source: PLoS One. 2025 Dec 1;20(12):e0335844. doi: 10.1371/journal.pone.0335844 (PMC12668615; doi:10.1371/journal.pone.0335844)
Supplement: S3 File — (PDF) [file pone.0335844.s003.pdf]

Oct 24, 2025

In vitro cultivation

In 1 collection

DOI
dx.doi.org/10.17504/protocols.io.8epv52wn4v1b/v1

Philip Laric[1]

[1]Department of veterinary science, LMU Munich, 81377, Germany

Philip Laric
vetmed. department AG Sabass

Create & collaborate more with a free account

Edit and publish protocols, collaborate in communities, share insights through comments, and track progress with run records.

Create free account

OPEN ACCESS

DOI: https://dx.doi.org/10.17504/protocols.io.8epv52wn4v1b/v1

Protocol Citation: Philip Laric 2025. In vitro cultivation. **protocols.io** https://dx.doi.org/10.17504/protocols.io.8epv52wn4v1b/v1

License: This is an open access protocol distributed under the terms of the **Creative Commons Attribution License**, which permits unrestricted use, distribution, and reproduction in any medium, provided the original author and source are credited

Protocol status: Working

Created: February 20, 2025

Last Modified: October 24, 2025

Protocol Integer ID: 124077

Keywords: Stimulation, Cultivation, Homogenisation, in vitro, rumen, rumen simulation, rumen cultivation, cultivation of rumen bacteria, rumen bacteria, rumen fluid, cultivation this protocol, microbial fermentation dynamic, cultivation, anaerobic condition, controlled feeding, stimulant

Abstract

This protocol outlines the in vitro cultivation of rumen bacteria. Stock solutions are prepared, rumen fluid is collected and processed under anaerobic conditions, and the culture is incubated with controlled feeding and gas monitoring. Stimulants are added at specific intervals to study microbial fermentation dynamics.

Materials

Reagents

- $(NH_4)_2SO_4$ (≥99,5%, Carl Roth, Karlsruhe, Germany)
- Acetic acid (≥99%, Carl Roth, Karlsruhe, Germany)
- Butyric acid (p.s., Sigma-Aldrich, Steinheim, Germany)
- $CaCl_2$ (≥98%, Carl Roth, Karlsruhe, Germany)
- Cellobiose (≥98%, Carl Roth, Karlsruhe, Germany)
- CO_2 (2.5, Linde, Pullach, Germany)
- DL-2-Methyl butyric acid (≥98%, Thermo Fisher Scientific, Waltham, Massachusetts, USA)
- Glucose (≥98%, Carl Roth, Karlsruhe, Germany)
- Glycerol (≥99,5%, Sigma-Aldrich, Steinheim, Germany)
- H_2O desalted (dH_2O) (GENO-OSMO-X, Grünbeck, Höchstädt, Germany)
- HCl (37%, Carl Roth, Karlsruhe, Germany)
- Hemin chloride (≥98%, Carl Roth, Karlsruhe, Germany)
- High-fiber silage (Ströh, Hamburg, Germany)
- iso-Butyric acid (≥99%, Carl Roth, Karlsruhe, Germany)
- iso-Valeric acid (p.s., Sigma-Aldrich, Steinheim, Germany)
- K_2HPO_4 (≥98%, Sigma-Aldrich, Steinheim, Germany)
- KCl (≥99%, Carl Roth, Karlsruhe, Germany)
- KH_2PO_4 (p.a., Carl Roth, Karlsruhe, Germany)
- KOH (≥85%, Carl Roth, Karlsruhe, Germany)
- L-cysteine-HCl * H_2O (≥98,5%, Carl Roth, Karlsruhe, Germany)
- Maltose (≥97%, Carl Roth, Karlsruhe, Germany)
- $MgCl_2$ (≥99%, Carl Roth, Karlsruhe, Germany)
- $MgSO_4$ (≥99%, Carl Roth, Karlsruhe, Germany)
- n-Valeric acid (≥99%, Thermo Fisher Scientific, Waltham, Massachusetts, USA)
- Na_2CO_3 (VWR, Darmstadt, Germany)
- Na_2HPO_4 (≥99%, Carl Roth, Karlsruhe, Germany)
- $Na_2S * 9 H_2O$ (≥98%, Thermo Fisher Scientific, Waltham, Massachusetts, USA)
- NaCl (≥99,5%, Carl Roth, Karlsruhe, Germany)
- $NaHCO_3$ (≥99,5%, Carl Roth, Karlsruhe, Germany)
- NaOH (≥98%, Carl Roth, Karlsruhe, Germany)
- NaOH (p.a., Carl Roth, Karlsruhe, Germany)
- Propionic acid (≥99%, VWR, Darmstadt, Germany)
- Resazurin sodium salt (Alfa Aesar, Haverhill, Massachusetts, USA)
- Starch, soluble (p.a., Carl Roth, Karlsruhe, Germany)
- Yeast extract (for cellculture, Carl Roth, Karlsruhe, Germany)

Equipments

- 1 L Erlenmeyer (DWK Life Sciences, Mainz, Germany)
- 10 L Polypropylene bucket (VWR, Darmstadt, Germany)

- Balloons (Latex, various, Amazon)
- Blender 2000W (Homegeek, Huanan, China)
- Boning knife (F. Dick, Deizisau, Germany)
- Bottle top filters Ø 0.22 µm (VWR, Darmstadt, Germany)
- Cheesecloth (Päckärdi, Huddersfield, UK)
- Cool packs (various)
- Fabric hose 8 mm (ESSKA, Hamburg, Germany)
- Immersion blender (Philips 5000 series, Philips, Amsterdam, Netherlands)
- Incubation hood (Medium, neoLab, Heidelberg, Germany)
- Ladle (IKEA, Delft, Netherlands)
- Orbital shaker (WS-1500, Wiggens, Straubenhardt, Germany)
- pH Meter (accumet AE150, Fisher Scientific, Schwerte, Germany)
- Polypropylene bucket 1 L (Berry Global, Evansville, USA)
- Sieve 1 mm mesh (IKEA, Delft, Netherlands)
- Syringe filter 0.22 µm (TPP, Trasadingen, Switzerland)
- Syringes 100 mL (Romed, Wilnis, Netherlands)
- Vaseline (Heinrich Hagner GmbH, Freudenstadt, Germany)
- Water bath (WB-12, Phoenix Instrument, Garbsen, Germany)
- Long gloves (Polyurethane, various, Amazon)

Stimulants:

- 3-nitrooxypropanol (3-NOP) (≥99%, synthesised and verified by Ewa Wegrzyn, Chemical and Pharmaceutical Faculty, LMU-Munich, Butenandtstraße 5, 81377 Munich)
- Bromoform (BF) (≥99%, Merck, Darmstadt, Germany)
- Ethanol (≥99,8%, Carl Roth, Karlsruhe, Germany)
- Freeze-dried *Asparagopsis taxiformis* (AT) powder (4.5 mg BF/g powder, Volta-Greentech, Solna, Sweden)
- Iodoform (IF) (99%, Thermo Fischer, Massachusetts, USA)
- Rapeseed oil (native, Alnatura GmbH, Darmstadt, Germany)

Troubleshooting

Reagent setup: In vitro cultivation

1 **Cellobiose stock:** Dissolve 5 g of cellobiose in 100 mL dH_2O and sterilise by filtration. Store at Room temperature for several months.

2 **Cysteine-HCl stock:** Dissolve 1 g of L-cysteine-HCl * H_2O in 50 mL of dH_2O and sterilise by filtration. Store at Room temperature for several months.

3 **Glucose stock:** Dissolve 20 g of glucose in 100 mL of dH_2O and sterilise by filtration. Store at Room temperature for several months.

4 **Hemin solution:** Dissolve 50 mg of Hemin chlorid in 1 mL [M] 1 Molarity (M) NaOH and fill up to 100 mL with dH_2O. Store at 4 °C - 8 °C for several months.

5 **Maltose stock:** Dissolve 20 g of maltose in 100 mL of dH_2O and sterilise by filtration. Store at Room temperature for several months.

6 **Mineral Solution:** Dissolve 3 g of KH_2PO_4, 6 g of NaCl, 3 g of $(NH_4)_2SO_4$, 0.6 g of $CaCl_2$ and 0.61 g of $MgSO_4$ in 1 L of dH_2O and sterilise by filtration. Store at Room temperature for several months.

> **Note**
>
> For stability, add 1-2 drops of HCl.

7 **Na-resazurin solution:** Dissolve 10 mg of Na-resazurin in 10 mL dH_2O and sterilise by filtration.

8 **Na_2S solution:** Dissolve 8.5 g of $Na_2S * 9 H_2O$ in 85 mL of dH_2O and dispense into 8.5 mL aliquots. Store in a tightly closed container at -20 °C.

9 **Rumen bacteria medium:** Prepare the rumen bacteria medium by dissolving 0.3 g of K_2HPO_4, 0.5 g of yeast extract, 0.5 g of Glycerol, 0.5 g of starch soluble, 38 mL of mineral solution, 1.6 mL of Na-resazurin solution, 3.1 mL of volatile fatty acids solution and 2 mL of hemin solution in 926.4 mL of dH_2O.

- Autoclave prior to completion with 2.5 mL of glucose stock, 2.5 mL of maltose stock, 10 mL of cellobiose stock, 12.5 mL of L-cysteine stock, 4 g of Na_2CO_3 and 2.5 mL of Na_2S stock. If necessary, adjust the pH of the complete media with KOH or by sparging with CO_2 to 6.7 - 6.8 [1].

10 **Saliva buffer:** Dissolve 22.2 mg of $CaCl_2$, 28.58 mg of $MgCl_2$, 9.8 g of $NaHCO_3$, 4.63 g of Na_2HPO_4, 0.47 g of NaCl and 0.57 g of KCl. If necessary adjust the pH to 8.2 using NaOH or by sparging with CO_2 [2].

11 **Volatile fatty acids solution:** Mix together 548.5 mL of acetic acid, 193.5 mL of propionic acid, 129 mL of butyric acid, 32.25 mL of isobutyric acid, 32.25 mL of DL-2-methylbutyric acid, 32.25 mL of valeric acid and 32.25 mL of isovaleric acid. Store at Room temperature .

Preparations

2m

12

2m

Note

The setup was usually cultivated for 11 days with one stimulation on day four. As a pilot experiment the setup was cultivated for 23 days with a second stimulation on day 16.

Dry the silage and blend it for 00:02:00 . Then sieve it through a 1 mm mesh and return the residues to the blender.

13 Prepare two feeding syringes.

14 Cut off the syringe tip but leave 1 cm intact.

15 Take a 50 mL serological pipette and cut the tip to fit your silicone tubes.

16 Deburr the edges with a cutter knife.

17 Trim the other end to expose the full pipette width.

18 3D-print a cylindrical mold and fix it to the syringe using hot glue.

19 Balance the serological pipette on the syringe and cast epoxy into the mold.

20 Check for leakage.

Sample collection

12m 30s

21 Fill the 1 L bucket with tap water and prewarm it with the cool packs to 55 °C the day prior to the slaughterhouse visit.

22 Pack a bag with a big 1 mm mesh sieve, a boning knife, several cheesecloths, an immersion blender, a ladle, a balloon attached to a valve filled with N_2 Gas, a serological pipette, that fits to the balloon valve, two 10 L buckets and long rubber gloves.

23 In the morning of the slaughterhouse visit, complete the rumen media and put it into the water bath at 39 °C , weigh 2 g of ground silage and add 10 ceramic loops per culture bottle. Place the bottles into the incubator at 39 °C .

24 Pack the bucket with the cool packs into a styrofoam box, take the prepared bag and go to the slaughterhouse.

25 At the slaughterhouse collect rumen fluid and content right after evisceration, through an incision of the rumen of four randomly selected cattle.

26 Blend the extracted liquid with some solids at maximum speed for 00:00:30 with the immersion blender [3]. 30s

27 Strain the rumen fluid through the sieve and squeeze it through two layers of cheesecloth into the prepared 1 L bucket.

28 Gently bubble N_2 through the rumen fluid for as long as the balloon allows and close the bucket afterwards.

29 Put the bucket into the styrofoam box filled with the warm cool packs and transport it back to the lab.

30 Prepare the inoculum by adding one part of rumen fluid to four parts of rumen media [1].

31 Pour the culture into the prewarmed culture bottles and close them with the assembled screwcaps.

32 Purge the culture with pure N_2 for 00:02:00 . 2m

33 Put the bottles into the orbital shaker under the incubation hood and attach the gas bags.

34 Set the orbital shaker to an intermittent shaking program of 00:05:00 shaking followed by 00:05:00 of pause between the measurements and set the incubation hood to temperature. 10m

Daily feeding

35 About half an hour prior to the feeding, weigh in 2 g of ground silage per culture, add 50 mL of saliva buffer [2] per culture and gently bubble it with nitrogen under vigorous stirring.

36 Take the bottles out of the shaker and incubation hood one by one.

37 Remove the plug, swirl once and immediately withdraw 50 mL of culture and either take samples
or discard them.

38 With the other pipette draw 50 mL of the feeding mix and inject it into the flask.

39 Insert the plug, exchange the gas bag and put the bottle back into the incubator.

Stimulation

40 Prepare the stimulants the day before stimulation.

Note

It is best to dissolve the stimulant, as it facilitates the handling. For the addition of solids, remove the bottom of a 1.5 mL Eppendorf tube and cover it with parafilm, then weigh in the stimulant. Now the Eppendorf tube can be attached to the silicon tube and the feeding solution can be flushed through the Eppendorf tube.

41 Stimulate the cultures on the fourth day of cultivation with 0.5 mL to 1.5 mL of liquid stimulant stock solution or any amount of solid stimulant.

42 Add the compounds prior to the injection of the feeding mix to wash all of the stimulant in.

Protocol references

[1] DSMZ. 330: Rumen Bacteria Medium. Deutsche Sammlung von Mikroorganismen und Zellkulturen GmbH. 2022 [cited 2025 Feb 27]. Available from: https://www.dsmz.de
[2] McDougall EI. Studies on ruminant saliva. 1. The composition and output of sheep's saliva. Biochem J. 1948;43(1):99–109
[3] Machado L, Magnusson M, Paul NA, Nys R de, Tomkins N. Effects of Marine and Freshwater Macroalgae on In Vitro Total Gas and Methane Production. PLOS ONE. 2014 Jan 22;9(1):e85289.
