# Supplementary material for: A reliable in vitro rumen culture system and workflow for screening anti-methanogenic compounds
Source: PLoS One. 2025 Dec 1;20(12):e0335844. doi: 10.1371/journal.pone.0335844 (PMC12668615; doi:10.1371/journal.pone.0335844)
Supplement: S4 File — (PDF) [file pone.0335844.s004.pdf]

Oct 24, 2025

Gas measurements

In 1 collection

DOI
dx.doi.org/10.17504/protocols.io.5jyl8d3e7g2w/v1

Philip Laric[1]

[1]Department of veterinary science, LMU Munich, 81377, Germany

Philip Laric
vetmed. department AG Sabass

Create & collaborate more with a free account

Edit and publish protocols, collaborate in communities, share insights through comments, and track progress with run records.

Create free account

OPEN ACCESS

DOI: https://dx.doi.org/10.17504/protocols.io.5jyl8d3e7g2w/v1

Protocol Citation: Philip Laric 2025. Gas measurements. **protocols.io** https://dx.doi.org/10.17504/protocols.io.5jyl8d3e7g2w/v1

License: This is an open access protocol distributed under the terms of the **Creative Commons Attribution License**, which permits unrestricted use, distribution, and reproduction in any medium, provided the original author and source are credited

Protocol status: Working

Created: February 19, 2025

Last Modified: October 24, 2025

Protocol Integer ID: 124076

Keywords: Calibration, Spectrophotometer, Gas measurement, Methane, CH4, Carbon dioxide, CO2, FTIR, Rumen, Gas production, methane production, gas measurement, gas measurements this method, using ftir spectroscopy, gas sample, calibration gas, ftir spectroscopy, gas composition, sealed gas system, recorded spectra, using recorded spectra

Abstract

This method describes gas measurement using FTIR spectroscopy. A sealed gas system is assembled, and calibration gases are prepared using syringes. Gas samples are collected, analyzed, and quantified using recorded spectra to evaluate gas composition and production.

Materials

Reagents

- CH_4 2.5% in N_2 (ALL-IN-GAS, Munich, Germany)
- CH_4 30% in N_2 (ALL-IN-GAS, Munich, Germany)
- CO_2 (2.5, Linde, Pullach, Germany)
- N_2 (5.0, Linde, Pullach, Germany)

Equipment

- 3-way connector 7 mm (OBI, Wermelskirchen, Germany)
- Fourier-transform infrared (FT-IR) spektrophotometer (SpektrumTwo, Perkin Elmer, Massachusetts, USA)
- Gas cuvette (Storm10 cm pyrex, Specac Ltd., UK)
- Needles 0.80 × 120 mm (Sterican®, B.Braun, Melsungen, Germany)
- Reservoir bottle with cap 150 mL (VWR, Darmstadt, Germany)
- Rubber stoppers 5×9×20 (Carl Roth, Karlsruhe, Germany)
- Syringes 200 and 300 mL (Romed, Wilnis, Netherlands)
- Windows for the gas cuvette (CaF_2 Window-Pair, Specac Ltd., UK)

Software

- Spectrum (Perkin Elmer, Massachusetts, USA)
- Spectrum Quant (Perkin Elmer, Massachusetts, USA)

Troubleshooting

FT-IR assembly

1 Set up the spectrophotometer and the gas cuvette according to the manual.

2 Mill a sealing ring from a rubber stopper for the 3-way connector and insert both into the inlet position.

3 Repeat this process for the female luer-lock adapter and the rubber stopper at the outlet position.

4 Attach one luer-lock valve to each opening and check if the system is airtight.

> **Note**
>
> One luer-lock valve on the 3-way connector is for the N_2 supply, while the other one serves as the injection port and N_2 tap.

5 Take the reservoir bottle and drill two holes into the screwcap. The holes should be large enough to be used as the inlet and outlet for the silicone tubing.

6 Attach a luer-lock valve to a short piece of silicone tube via a male luer-lock adaptor.

7 Pierce a rubber stopper with several needles, cut off the luer-lock adaptors of the needles and plug them into the open end of the silicone tubing.

> **Note**
>
> Make sure the cuts do not obstruct the canules.

8 Secure the tube with the needles to one of the hole in the screwcap. The needles should reach to the bottom of the flask.

9 Attach another tube to the second hole. 3D-Print a cylindrical mold and glue it to the screwcap. Pour epoxy into the mold and let it cure.

10 Fill the bottle with tap water until all needles are submerged.

11 Direct the exhaust tube to a ventilation pipe.

Syringe assembly

12 Take a 200 mL and a 300 mL syringe and cut off their tips, so that the female end of a luer-lock valve fits into the hole and does not obstruct the syringe piston.

13 3D-Print two cylindrical sections that leave room for the valve movement and have sufficient attachment area.

14 Seal the female ends of the luer-lock valves with vaseline and apply vaseline to the syringe piston as well.

15 Insert the luer-lock valve into the syringe tip and move the piston to the exhaust position.

16 Hot-glue the mold into position, cast the epoxy and let it cure.

Note

Move the valve to the closed position to prevent obstruction of the valve by the epoxy.

17 Check for leakage, by submerging in water and applying pressure.

Generation of the calibration curve for N_2 and CO_2

18 Start the FT-IR spectrophotometer and the spectrum software. Insert the assembled gas cuvette into the lightpath and attach the N_2 gas line to the gas inlet of the cuvette.

19 Flush the gas cuvette and record the background spectrum.

20 Measure at least three spectra for each concentration.

Note

To prepare diluted gas standards, draw calibration gas into the 300-mL syringe. Adjust the volume based on the dilution factor. Exhaust the gas through a needle into a water-filled vessel and wait for the pressure equilibration to precisely measure the volume. Then take the 200-mL syringe and draw the needed amount of N_2 gas to fill the 300-mL syringe to 300 mL. Transfer the gas into the 300-mL syringe with an female/female adaptor.

21 Save the spectra and load them into the spectrum quant software to generate the quantification method according to the manual.

22 Load the generated methods into the spectrum software.

Evaluation of the in vitro gas production

23 After the feeding, close both luer-lock valves and replace the gas bag.

Note

The gas bags can now be stored until evaluation at either room temperature or at -20 °C .

24 Briefly freeze the filled gas bags to reduce the gas humidity.

25 Start the spectrum software, purge the gas cuvette with N_2 and record the background spectrum.

26 Withdraw 300 mL of gas from the gas bags into the 300-mL syringe.

Note

If gas production is less than 300 mL, use the next 50-mL step and compensate for the missing volume with N_2.

27 Measure the residual gas content of the gas bag with the 200-mL syringe and record the total gas production.

28 Inject 300 mL of gas into the gas cuvette via the injection port.

29 Wait for the pressure to equilibrate and record the measurement.

30 After all samples are measured, run the evaluation methods to quantify the gas contents.

31 Proceed with data evaluation.
