# Supplementary material for: A reliable in vitro rumen culture system and workflow for screening anti-methanogenic compounds
Source: PLoS One. 2025 Dec 1;20(12):e0335844. doi: 10.1371/journal.pone.0335844 (PMC12668615; doi:10.1371/journal.pone.0335844)
Supplement: S5 File — (PDF) [file pone.0335844.s005.pdf]

Oct 24, 2025 Version 1

Microbiome analysis V.1

In 1 collection

DOI
dx.doi.org/10.17504/protocols.io.261gerqeyl47/v1

Philip Laric[1]

[1]Department of veterinary science, LMU Munich, 81377, Germany

Philip Laric
vetmed. department AG Sabass

Create & collaborate more with a free account

Edit and publish protocols, collaborate in communities, share insights through comments, and track progress with run records.

Create free account

OPEN ACCESS

DOI: https://dx.doi.org/10.17504/protocols.io.261gerqeyl47/v1

Protocol Citation: Philip Laric 2025. Microbiome analysis. **protocols.io**
https://dx.doi.org/10.17504/protocols.io.261gerqeyl47/v1

License: This is an open access protocol distributed under the terms of the **Creative Commons Attribution License**, which permits unrestricted use, distribution, and reproduction in any medium, provided the original author and source are credited

Protocol status: Working

Created: February 20, 2025

Last Modified: October 24, 2025

Protocol Integer ID: 124078

Keywords: Amplification, PCR, Nucleic acid, In vitro, rumen, rumen simulation, microbiome, microbiome analysis, rumen PCR, microbiota, rumen microbiota, microorganisms, rumen microorganisms, microbiome analysis of ruminal, microbiome analysis, microbiome analysis this protocol, rumen sample, microbiome, ruminal, microbial community composition, dna extraction, rrna gene, purified dna, amplicon purification, pcr amplification

Abstract

This protocol describes the microbiome analysis of ruminal or *in vitro* rumen samples. It includes sample collection, DNA extraction using bead-beating and column purification, PCR amplification of the 16S rRNA gene, and amplicon purification. The purified DNA is quantified and sent for sequencing to assess microbial community composition.

Materials

Reagents

- Agarose-LE (Biozym Scientific, Oldendorf, Germany)
- AMPure XP Beads (Beckmann Coulter, Brea, California, USA)
- CH_3COOH (≥99%, Carl Roth, Karlsruhe, Germany)
- Deoxynucleotide solution (New England Biolabs, Frankfurt am Main, Germany)
- DNA extraction kit (ExtractME, BLIRT S.A., Gdańsk, Poland)
- DNA ladder 1 kb (New England Biolabs, Frankfurt am Main, Germany)
- EDTA (Merck, Darmstadt, Germany)
- Ethanol (≥99,5%, Carl Roth, Karlsruhe, Germany)
- Gel loading dye, blue 6x (New England Biolabs, Frankfurt am Main, Germany)
- DNA ladder 100 bp (New England Biolabs, Frankfurt am Main, Germany)
- GelRed® Nucleic acid stain 10000x (New England Biolabs, Frankfurt am Main, Germany)
- Isopropanol (≥99,8%, Sigma-Aldrich, Steinheim, Germany)
- NaCl (≥99,5%, Carl Roth, Karlsruhe, Germany)
- NH_4CH_3COO (≥99%, Carl Roth, Karlsruhe, Germany)
- Q5® High-fidelity DNA polymerase (New England Biolabs, Frankfurt am Main, Germany)
- SDS (≥99%, Carl Roth, Karlsruhe, Germany)
- Tris-HCl (≥99%, Sigma-Aldrich, Steinheim, Germany)
- Tris-OH (≥99%, Sigma-Aldrich, Steinheim, Germany)
- 5x Q5 reaction buffer (New England Biolabs, Frankfurt am Main, Germany)
- deoxynucleotide solution mix 10 mM each (dNTP's) (New England Biolabs, Frankfurt am Main, Germany)
- ddH_2O (Sigma-Aldrich, Steinheim, Germany)
- 96-well PCR plate (Eppendorf, Hamburg, Germany)
- Reagent reservoir (VWR, Darmstadt, Germany)

Equipments

- Bead beater (Tissue Lyser MM300, QUIAGEN, Hilden, Germany)
- Centrifuge (Fresco™ 21 Microcentrifuge, Thermo Fisher Scientific, Waltham, Massachusetts, USA)
- Gel Caster (Bio-Rad Laboratories, Hercules, California, USA)
- Gel comb (10-Well comb, Bio-Rad Laboratories, Hercules, California, USA)
- Gel documentation system (Vilber Lourmat, Biovision 3000 WL, Eberhardzell, Germany)
- Gel tray (7×8 cm UV-transparent mini-gel tray, Bio-Rad Laboratories, Hercules, California, USA)
- Glass beads 0.1 mm (Carl Roth, Karlsruhe, Germany)
- Horizontal electrophoresis system (Mini-Sub Cell GT Cell, Bio-Rad Laboratories, Hercules, California, USA)
- Imaging chamber (Darkroom – CN3000, PEQLAB, Erlangen, Germany)
- Neodymium magnets (EarthMag, Dortmund, Germany)
- PCR tube strips (Eppendorf, Hamburg, Germany)
- Power supply (PowerPac™ Basic, Bio-Rad Laboratories, Hercules, California, USA)
- Rack for PCR tubes (neoLab, Heidelberg, Germany)
- Thermocycler (Vapo Protect Mastercycler® Pro, Eppendorf, Hamburg, Germany)
- ThermoMixer® C (Eppendorf, Hamburg, Germany)
- UV-Vis cuvettes (PMMA, VWR, Darmstadt, Germany)

- UV-Vis spectrophotometer (Nanodrop1000, Thermo Fisher Scientific, Waltham, Massachusetts, USA)
- Vortexer (Vortex-Genie™ 2, Scientific Industries, Bohemia, New York, USA)
- Zirconia beads 0.5 mm (Scientific Industries, Bohemia, New York, USA)

Software

- DADA2 package (v.1.14) **[70]**
- DNA-Quantification (Nanodrop1000, Thermo Fisher Scientific, Waltham, Massachusetts, USA)
- Gel visualisation (VISION-CAPT, Vilber Lourmat, Eberhardzell, Germany)
- R-Studio 2022.02.2+485 (PBC, Boston, USA)

External services

- Sequencing service (GENEWIZ Germany GmbH, Leipzig)

Troubleshooting

Preparations

1 Prepare the magnetic stand for the AMPure XP beads. Use hot glue to fix one magnet each in the north-south direction between two holes under the PCR rack.

2 Reagent setup:
Tris Buffer: Dissolve 89 mg of Tris-OH and 42 mg of Tris-HCl in 100 mL H_2O.

Sample collection

5m

3 From the daily feed waste, collect 2 × 2 mL of samples using a cut-off pipette tip to transfer both liquid and solid contents.

4 Separate the solids from the liquid by centrifugation step at 10000 x g, 4°C, 00:05:00 .

5m

5 Discard the supernatant and freeze the pellet at -80 °C until further processing.

Repeated bead-beating + Column [1]

1h 8m

6 Thaw the pellets On ice and add 0.3 g of 0.1 mm beads and 0.1 g of 0.5 mm beads.

7 Homogenize the pellets using a bead beater at maximum speed for 00:03:00 .

3m

8 Incubate at 70 °C for 00:15:00 , shaking gently every 5 minutes using a thermomixer.

15m

9 Centrifuge at 16000 x g, 4°C, 00:05:00 .

5m

10 Transfer the supernatant to a new Eppendorf tube.

Note

Optional: Add another 300 µL lysis buffer to the pellet and repeat steps 7-10 if needed.

11 Add 200 µL of [M] 10 Molarity (M) NH_4CH_3COO (260 µL if the optional step was carried out), mix well and incubate On ice for 00:05:00 . 5m

12 Centrifuge at 16000 x g, 4°C, 00:05:00 . 5m

13 Transfer 1 mL of the supernatant to a new 2 mL Eppendorf tube, add an equal volume of ice-cold isopropanol, mix well and incubate On ice for 00:30:00 . 30m

14 Centrifuge at 16000 x g, 4°C, 00:05:00 . 5m

15 Discard the supernatant, wash the nucleic acid pellet with ice-cold 70% ethanol and let it dry.

16 Dissolve the nucleic acid pellet in 150 µL of elution buffer from the kit.

Removal of RNA, Protein and Purification (Blirt ExtractMe Kit) 1h 36m

17 Add 1.5 µL of DNase-free RNase and incubate at 37 °C for 00:15:00 . 15m

18 Add 15 µL of proteinase K and 250 µL of Buffer GL, mix well, and incubate at 55 °C for 00:10:00 . Vortex every 60s. 10m

19 Add 400 µL of GB-Buffer, mix well and spin down any residues by centrifuging at 16000 x g, 00:01:00 . Transfer the supernatant to an ExtractMe column and centrifuge at 16000 x g, 00:01:00 . 2m

20 Discard the flow-through. Take a new tube, add 600 µL of Buffer GW1 and centrifuge at 15000 x g, Room temperature, 00:01:00 . 1m

21 Discard the flow-through, add 500 µL of Buffer GW2 and centrifuge at 15000 x g, Room temperature, 00:01:00 . 1m

22 Discard the flow-through and dry the column by centrifugation at 15000 x g, Room temperature, 00:01:00 . 1m

23 Transfer the column to a new Eppendorf tube. Add 200 µL of warm Elution Buffer and incubate at Room temperature for 00:05:00 . 5m

24 Centrifuge at 15000 x g, Room temperature, 00:01:00 to elute the DNA. 1m

25 Prepare an agarose gel by boiling 0.4 g of agarose in 50 mL TAE Buffer.

> **Note**
>
> Refill the evaporated water.

26 Add 1 µL of GelRed, pour the gel into the mold, insert the comb and let it cool.

27 Prepare the samples by mixing 5 parts of DNA Sample with 1 part of gel loading dye.

28 Load 2 µL of each sample per well and include 5 µL of the 1 kbp ladder in each run.

29 Run the gel at 80 V for 01:00:00 . 1h

Amplicon PCR [2]

9m 30s

30 Prepare a PCR-Mastermix:

	A	B
	5x Q5 reaction buffer	10 µL

	A	B
	10 mM dNTP's	1 µL
	Forward primer	2.5 µL
	Reverse primer	2.5 µL
	Q5 HiFi DNA polymerase	0.5 µL
	ddH2O	28.5 µL
	One reaction	40 µL

31 Pipette 40 µL Mastermix into every PCR tube/well, then add 2 µL of template.

32 For the amplification of the 16S small subunit ribosomal RNA fragments following primers were used:

	A	B	C
		**Sequence**	**Reference**
	Forward Primer	5'-ACACTCTTTCCCTACACGACGCTCTTCCGATCT GTGYCAGCMGCCGCGGTAA -3'	[3]
	Reverse Primer	5'-GACTGGAGTTCAGACGTGTGCTCTTCCGATCT GGACTACNVGGGTWTCTAAT -3'	[4]

Table 1: List of primers used for amplification of the 16S small subunit ribosomal RNA fragment and subsequent sequencing. The primers are annotated in 5' - 3' direction. The first row is the sequencing adaptor; The second line is the degenerated binding sequence.

33 Seal the individual reaction tubes and perform the PCR using the following settings:

9m 30s

- 95 °C for 00:03:00
- 35 cycles of:

1. 95 °C for 00:00:30
2. 55 °C for 00:00:30
3. 72 °C for 00:00:30

- 72 °C for 00:05:00
- Hold at 4 °C

PCR purification

28m

34 Centrifuge the amplicon PCR plate at 1000 x g, 20°C, 00:01:00 to collect the condensate, then carefully remove the seal. 11m

- Allow the beads to air-dry for 00:10:00 or until all ethanol has evaporated.

35 Vortex the AMPure XP beads for 00:00:30 to ensure an even dispersion. Then, transfer the appropriate volume of bead solution into a reagent reservoir based on the number of samples being processed. 30s

36 Using a multichannel pipette, add 20 µL of AMPure XP beads to each well of the amplicon PCR plate.

37 Seal the plate and shake at 1800 rpm, Room temperature , 00:02:00 in the Thermomixer. 2m

38 Incubate at Room temperature without shaking for 00:05:00 . 5m

39 Place the plate on a magnetic stand for 00:02:00 or until the supernatant has cleared. 2m

40 **With the amplicon PCR plate on the magnetic stand perform an ethanol wash:** 30s

- Use a multichannel pipette to remove and discard the supernatant.

> Note
>
> Be careful not to remove any beads. Wash the beads with freshly prepared 80% ethanol:

- Using a multichannel pipette, add 200 µL of freshly prepared 80% ethanol to each sample well.
- Incubate the plate on the magnetic stand for 00:00:30 .
- Carefully remove and discard the supernatant.

41 **Perform a second ethanol wash:** 30s

- Using a multichannel pipette, add 200 µL of freshly prepared 80% ethanol to each sample well.
- Incubate the plate on the magnetic stand for 00:00:30 .
- Carefully remove and discard the supernatant.

42 Remove the amplicon PCR plate from the magnetic stand. Using a multichannel pipette, add 52.5 µL of [M] 10 millimolar (mM) Tris pH 8.5 to each well of the amplicon PCR plate.

43 Seal the plate and shake at 1800 rpm, Room temperature , 00:02:00 , ensuring the beads are fully resuspended. 2m

44 Incubate at Room temperature for 00:02:00 . 2m

45 Place the plate on the magnetic stand for 00:02:00 or until the supernatant has cleared. 2m

46 Using a multichannel pipette, carefully transfer 50 µL of the supernatant from the amplicon PCR plate to a new 96-well PCR plate.

Note

Ensure no beads are transferred.

47 Measure the concentration using Nanodrop1000 and dilute the samples to 20 ng/µL as needed.

48 Send the tubes to an external sequencing service and await the data.

Protocol references

[1] Yu Z, Morrison M. Improved extraction of PCR-quality community DNA from digesta and fecal samples. BioTechniques. 2004 May;36(5):808–12
[2] 16S Illumina Amplicon Protocol : earthmicrobiome [Internet]. 2022 [cited 2022 May 13]. Available from: https://earthmicrobiome.org/protocols-and-standards/16s/
[3] Parada AE, Needham DM, Fuhrman JA. Every base matters: assessing small subunit rRNA primers for marine microbiomes with mock communities, time series and global field samples. Environ Microbiol. 016;18(5):1403–14
[4] Apprill A, McNally S, Parsons R, Weber L. Minor revision to V4 region SSU rRNA 806R gene primer greatly increases detection of SAR11 bacterioplankton. Aquat Microb Ecol. 2015 Jun 4;75(2):129–37
