# Supplementary material for: A reliable in vitro rumen culture system and workflow for screening anti-methanogenic compounds
Source: PLoS One. 2025 Dec 1;20(12):e0335844. doi: 10.1371/journal.pone.0335844 (PMC12668615; doi:10.1371/journal.pone.0335844)
Supplement: S6 File — (PDF) [file pone.0335844.s006.pdf]

Oct 24, 2025

Data evaluation

In 1 collection

DOI
dx.doi.org/10.17504/protocols.io.81wgbrm41lpk/v1

Philip Laric[1]

[1]Department of veterinary science, LMU Munich, 81377, Germany

Philip Laric
vetmed. department AG Sabass

Create & collaborate more with a free account

Edit and publish protocols, collaborate in communities, share insights through comments, and track progress with run records.

Create free account

OPEN ACCESS

DOI: https://dx.doi.org/10.17504/protocols.io.81wgbrm41lpk/v1

Protocol Citation: Philip Laric 2025. Data evaluation. **protocols.io** https://dx.doi.org/10.17504/protocols.io.81wgbrm41lpk/v1

License: This is an open access protocol distributed under the terms of the **Creative Commons Attribution License**, which permits unrestricted use, distribution, and reproduction in any medium, provided the original author and source are credited

Protocol status: Working

Created: March 06, 2025

Last Modified: October 24, 2025

Protocol Integer ID: 124081

Keywords: Protein visualisation , Microbiome analysis, bioinformatics, metagenomics, in vitro, rumen simulation, rumen, microbiome data, microbiome, gas production data, sequencing analysis, grubbs test, outlier detection, bioinformatics method, data evaluation, taxonomic classification via the silva database, taxonomic classification, gas measurement, data evaluation this data evaluation process, data evaluation process, robust interpretation of both dataset, using dada2

Abstract

This data evaluation process integrates gas measurement and microbiome sequencing analysis through statistical and bioinformatics methods. Gas production data is processed for outlier detection using the Grubbs test, with significance determined by Student's t-test. Microbiome data undergoes quality control, filtering, and dereplication using DADA2 in R, followed by taxonomic classification via the SILVA database. Statistical analyses and visualizations are performed to assess significant differences across groups, ensuring robust interpretation of both datasets.

Troubleshooting

General data evaluation

1 Process the raw data in Libre Office calculator.

- Perform a Grubbs test to identify outliers. Values with two or more consecutive outliers are excluded from further analysis.
- For evaluating the variances and significance levels, perform a one- or two-sided Student's t-test, depending on the datasets [1].

2 Reject H_0 if two or more consecutive time points show significant differences (alpha = 0.05).

3 Create graphs for the data in GraphPad PRISM 5. Edit the Figures in Inkscape 1.2.

4 Perform the protein visualisation using ChimeraX [2].

Microbiome data analysis

5 For Linux: Install conda from terminal once

```
wget https://repo.anaconda.com/miniconda/Miniconda3-latest-Linux-
x86_64.sh
```

```
bash Miniconda3-latest-Linux-x86_64.sh
```

6 Create the working environment from terminal:

```
conda install -y -c conda-forge mamba
```

```
mamba create -y -n hb-dada2-ex-wf -c conda-forge -c bioconda -c
defaults \
cutadapt r-base r-tidyverse r-vegan r-dendextend r-viridis \
bioconductor-phyloseq bioconductor-deseq2 bioconductor-dada2 \
bioconductor-decipher bioconductor-decontam r-biocmanager \
r-matrix libopenblas
```

```
conda activate hb-dada2-ex-wf
```

7 Start RStudios from terminal:

```
rstudio
```

8 From the terminal in RStudios go to your desired working directory:

```
cd ~/Desktop/16SSequencing/Folder
```

9 Get the sequencing data from the Azenta website via sftp:

```
sftp credentials@gweusftp.brooks.com #
```

```
lcd ~/Desktop/16SSequencing/Folder/ #change download directory
```

```
ls #display files in your current location
```

```
get -r filename #download the folder with all files inside
```

```
exit
```

10

```
# cd ~/Desktop/16SSequencing/Folder/raw_fastq #go to your data
```

```
gunzip -v *gz #unpack your data
```

```
ls *_R1_001.fastq | cut -f1 -d "_" > samples #make a list of the
samples in samples
```

11

```
for sample in $(cat samples)

do

echo "On sample: $sample"

cutadapt -a ^GTGYCAGCMGCCGCGGTAA...ATTAGAWACCCBDGTAGTCC \
                -A ^GGACTACHVGGGTWTCTAAT...TTACCGCGGCKGCTGRCAC \
                -m 215 -M 285 --discard-untrimmed \
                -o ${sample}_R1_trimmed.fastq -p
${sample}_R2_trimmed.fastq \
                 ${sample}_R1_001.fastq ${sample}_R2_001.fastq \
                 >> cutadapt_primer_trimming_stats.txt 2>&1

done  #remove the primers of the forward and reverse reads
```

12

```
# paste <(ls *_R1_001.fastq | cut -f1 -d "_") <(grep "passing"
cutadapt_primer_trimming_stats.txt | cut -f3 -d "(" | tr -d ")")
<(grep "filtered" cutadapt_primer_trimming_stats.txt | cut -f3 -d
"(" | tr -d ")") > samplespass #check the fraction of retained
base pairs
```

13 Switch to the RStudios console

```
library(dada2)
library(writexl) #initialize the libraries
```

```
setwd ("~/Desktop/16SSequencing/Folder")
```

```
samples <- scan("samples", what="character") #make a list of the
samples in R
```

```
forward_reads <- paste0(samples, "_R1_trimmed.fastq") #make a list
of the forward reads
reverse_reads <- paste0(samples, "_R2_trimmed.fastq") #make a list
of the reverse reads
```

```
filtered_forward_reads <- paste0(samples, "_R1_filtered.fastq")
#make a list of the forward reads for filtering
filtered_reverse_reads <- paste0(samples,
"_R2_filtered.fastq")#make a list of the reverse reads for
filtering
```

```
plotQualityProfile(forward_reads)
plotQualityProfile(reverse_reads) #display the quality score of
the reads over the length
```

14

```
filtered_out <- filterAndTrim(forward_reads,
filtered_forward_reads, reverse_reads,
                        filtered_reverse_reads, maxEE=c(2,2),
rm.phix=TRUE,
                        minLen=175, maxN = 0, verbose=TRUE,
truncLen=c(210,210))

# filter and trim the reads based on the arguments. maxEE
specifies the allowed erroneous bases for forward and reverse
reads. rm.phix removes reads that match the PhiX bacteriophage
genome. minLen sets the minimum read length. truncLen sets the
trimming length
```

15

```
plotQualityProfile(filtered_forward_reads)
plotQualityProfile(filtered_reverse_reads)
#display the quality score of the filtered reads over the length
```

16

```
err_forward_reads <- learnErrors(filtered_forward_reads,
multithread=TRUE, verbose=TRUE)
err_reverse_reads <- learnErrors(filtered_reverse_reads,
multithread=TRUE, verbose=TRUE)
# learn about the error-signature
```

17

```
plotErrors(err_forward_reads, nominalQ=TRUE)
plotErrors(err_reverse_reads, nominalQ=TRUE)
#plot the error signature
```

18

```
dada_forward_reads <- dada(filtered_forward_reads,
err=err_forward_reads, multithread=TRUE, verbose=TRUE, pool =
"pseudo")
dada_reverse_reads <- dada(filtered_reverse_reads,
err=err_reverse_reads, multithread=TRUE, verbose=TRUE, pool =
"pseudo")
#dereplicate reads
```

19

```
merged_amplicons <- mergePairs(dada_forward_reads,
filtered_forward_reads, dada_reverse_reads,
filtered_reverse_reads, trimOverhang=TRUE, minOverlap=160,
verbose=TRUE)
#merge forward and reverse reads. minOverlap specifies minimal
overlap in bps.
```

20

```
seqtab <- makeSequenceTable(merged_amplicons)
```

```
seqtab.nochim <- removeBimeraDenovo(seqtab, verbose=T) #remove
chimeras
```

```
getN <- function(x) sum(getUniques(x))
```

21

```
summary_tab <- data.frame(row.names=samples,
dada2_input=filtered_out[,1],
                            filtered=filtered_out[,2],
dada_f=sapply(dada_forward_reads, getN),
                            dada_r=sapply(dada_reverse_reads,
getN),
                            merged=sapply(merged_amplicons, getN),
nonchim=rowSums(seqtab.nochim),

perc_reads_retained=round(rowSums(seqtab.nochim)/filtered_out[,1]*
100, 1))
summary_tab #get an idea where reads are dropped, if you are left
with too few reads.
```

22

```
write_xlsx(summary_tab, "FOLDER_summary_tab.xlsx")
```

23 Download the SILVA v138 database once

```
download.file(url =
"http://www2.decipher.codes/Classification/TrainingSets/SILVA_SSU_
r138_2019.RData", destfile =
"~/Desktop/16SSequencing/SILVA_SSU_r138_2019.RData")
```

24 Initialize the database

```
library(DECIPHER)
```

```
load("~/Desktop/16SSequencing/SILVA_SSU_r138_2019.RData")
```

```
load("~/Desktop/16SSequencing/SILVA_SSU_r138_2019.RData")
```

```
ids <- IdTaxa(dna, trainingSet, strand="both", processors=NULL,
verbose=TRUE)
```

```
load("ids.RData")
```

25 Generating standard taxonomy goods

```
asv_seqs <- colnames(seqtab.nochim)
asv_headers <- vector(dim(seqtab.nochim)[2], mode="character")
for (i in 1:dim(seqtab.nochim)[2]) {asv_headers[i] <-
paste(">ASV", i, sep="_")}
asv_fasta <- c(rbind(asv_headers, asv_seqs))
write(asv_fasta, "ASVs.fa")
asv_tab <- t(seqtab.nochim)
row.names(asv_tab) <- sub(">", "", asv_headers)
write.table(asv_tab, "ASVs_counts.tsv", sep="\t", quote=F,
col.names=NA)
ranks <- c("domain", "phylum", "class", "order", "family",
"genus", "species")
asv_tax <- t(sapply(ids, function(x) {
                 m <- match(ranks, x$rank)
                 taxa <- x$taxon[m]
                 taxa[startsWith(taxa, "unclassified_")] <- NA
                 taxa }))
colnames(asv_tax) <- ranks
rownames(asv_tax) <- gsub(pattern=">", replacement="",
x=asv_headers)
write.table(asv_tax, "ASVs_taxonomy.tsv", sep = "\t", quote=F,
col.names=NA)
```

26 Removing contaminants, if blank or lab background samples were included

```
library(decontam)
colnames(asv_tab)
vector_for_decontam <- c(rep(TRUE, # blanks or lab background),
rep(FALSE, #samples))
contam_df <- isContaminant(t(asv_tab), neg=vector_for_decontam)
table(contam_df$contaminant)
contam_asvs <- row.names(contam_df[contam_df$contaminant == TRUE,
])
contam_indices <- which(asv_fasta %in% paste0(">", contam_asvs))
dont_want <- sort(c(contam_indices, contam_indices + 1))
asv_fasta_no_contam <- asv_fasta[- dont_want]
asv_tab_no_contam <- asv_tab[!row.names(asv_tab) %in% contam_asvs,
]
asv_tax_no_contam <- asv_tax[!row.names(asv_tax) %in% contam_asvs,
]
write(asv_fasta_no_contam, "ASVs-no-contam.fa")
write.table(asv_tab_no_contam, "ASVs_counts-no-contam.tsv",
sep="\t", quote=F, col.names=NA)
write.table(asv_tax_no_contam, "ASVs_taxonomy-no-contam.tsv",
sep="\t", quote=F, col.names=NA)
```

27 Load the libraries for the analysis and tidy up the workspace

```
library(tidyverse)
library(phyloseq)
library(vegan)
library(DESeq2)
library(dendextend)
library(viridis)
library("readxl")
library("ggplot2")
rm(list=ls())
```

28 Make an excel file with the columns: sample; day; compound; concentration; unit; color and complete it for your samples. Save it as sample_info.xls

29

```
count_tab <- read.table("ASVs_counts-no-contam.tsv", header=T,
row.names=1, check.names=F, sep="\t") [ , -c(#specify the blank or
lab background samples)]
names(count_tab) <- scan("samples", what="character", sep = NULL)
```

```
tax_tab <- as.matrix(read.table("ASVs_taxonomy-no-contam.tsv",
header=T, row.names=1, check.names=F, sep="\t"))
```

Format the sample_info

```
sample_info_tab <- read_xls("sample_info.xls", col_names = T,
col_types=NULL,na="",skip= 0)
sample_info_tab <-  as.data.frame(sample_info_tab)
row.names(sample_info_tab) <- sample_info_tab$sample
sample_info_tab <- sample_info_tab[,-1]
sample_info_tab$color <- as.character(sample_info_tab$color)
sample_info_tab$compound <-  as.factor(sample_info_tab$compound)
sample_rownames <- rownames(sample_info_tab)
sample_info_tab
```

```
sorted_colnames <- colnames(count_tab)[match(sample_rownames,
colnames(count_tab))]
count_tab <- count_tab[, sorted_colnames]
```

30 Taxonomic summaries

```
sample_info_tab_phy <- sample_data(sample_info_tab)
count_tab_phy <- otu_table(count_tab, taxa_are_rows=T)
tax_tab_phy <- tax_table(tax_tab)
ASV_physeq <- phyloseq(count_tab_phy, tax_tab_phy,
sample_info_tab_phy)
phyla_counts_tab <- otu_table(tax_glom(ASV_physeq,
taxrank="phylum"))
phyla_tax_vec <- as.vector(tax_table(tax_glom(ASV_physeq,
taxrank="phylum"))[,"phylum"])
rownames(phyla_counts_tab) <- as.vector(phyla_tax_vec)
```

Account for the unclassified counts

```
unclassified_tax_counts <- colSums(count_tab) -
colSums(phyla_counts_tab)
phyla_and_unidentified_counts_tab <- rbind(phyla_counts_tab,
"Unclassified"=unclassified_tax_counts)
```

Resolve the phylum of euryarchaeota by classes

```
temp_major_taxa_counts_tab <-
phyla_and_unidentified_counts_tab[!row.names(phyla_and_unidentifie
d_counts_tab) %in% "Euryarchaeota", ]
class_counts_tab <- otu_table(tax_glom(ASV_physeq,
taxrank="class"))
class_tax_phy_tab <- tax_table(tax_glom(ASV_physeq,
taxrank="class"))
phy_tmp_vec <- class_tax_phy_tab[,2]
class_tmp_vec <- class_tax_phy_tab[,3]
rows_tmp <- row.names(class_tax_phy_tab)
class_tax_tab <- data.frame("phylum"=phy_tmp_vec,
"class"=class_tmp_vec, row.names = rows_tmp)
eurya_classes_vec <- as.vector(class_tax_tab[class_tax_tab$phylum
== "Euryarchaeota", "class"])
rownames(class_counts_tab) <- as.vector(class_tax_tab$class)
eurya_class_counts_tab <-
class_counts_tab[row.names(class_counts_tab) %in%
eurya_classes_vec, ]
eurya_no_class_annotated_counts <-
phyla_and_unidentified_counts_tab[row.names(phyla_and_unidentified
_counts_tab) %in% "Euryarchaeota", ] -
colSums(eurya_class_counts_tab)
```

Combine the tables and generate a proportions table

```
major_taxa_counts_tab <- rbind(temp_major_taxa_counts_tab,
eurya_class_counts_tab,
"Unresolved_Euryarchaeota"=eurya_no_class_annotated_counts)
identical(colSums(major_taxa_counts_tab), colSums(count_tab))
major_taxa_proportions_tab <- apply(major_taxa_counts_tab, 2,
function(x) x/sum(x)*100)
```

Include a filtering threshold, but exclude the Methanogens(Euryarchaeota) from filtering

```
evading_filtering_tab <- rbind(major_taxa_proportions_tab)
evade_filtering <-
major_taxa_proportions_tab[row.names(major_taxa_proportions_tab)
%in% "Methanobacteria", ]
major_taxa_proportions_tab <-
evading_filtering_tab[!row.names(evading_filtering_tab) %in%
"Methanobacteria", ]
temp_filt_major_taxa_proportions_tab <-
data.frame(major_taxa_proportions_tab[apply(major_taxa_proportions
_tab, 1, max) > 5, ], check.names = FALSE)
temp_filt_major_taxa_proportions_tab <-
data.frame(major_taxa_proportions_tab, check.names = FALSE)
filtered_proportions <- colSums(major_taxa_proportions_tab) -
colSums(temp_filt_major_taxa_proportions_tab)
filt_major_taxa_proportions_tab <-
rbind(temp_filt_major_taxa_proportions_tab,
"Other"=filtered_proportions, "Methanobacteria"=evade_filtering)
library(writexl)
filt_major_taxa_proportions_tab$MajorTaxa <-
row.names(filt_major_taxa_proportions_tab)
filt_major_taxa_proportions_tab <-
filt_major_taxa_proportions_tab[ ,c("MajorTaxa",
names(filt_major_taxa_proportions_tab)[-
ncol(filt_major_taxa_proportions_tab)])]
write_xlsx(filt_major_taxa_proportions_tab,
"FOLDER_unfiltered.xlsx")
```

31 Open the excel file and do further analysis

- Perform a Mann-Whitney U-test to assess whether the stimulations are different from the negative control
- Generate and edit graphs

Protocol references

1. Welch BL. The Significance of the Difference between two Means when the Population Variances are unequal. Biometrika [Internet]. 1938 Feb 1 [cited 2023 May 2]; Available from: https://www.scinapse.io/papers/1973158119}
2. Pettersen EF, Goddard TD, Huang CC, Meng EC, Couch GS, Croll TI, et al. UCSF ChimeraX: Structure visualization for researchers, educators, and developers. Protein Sci Publ Protein Soc. 2021 Jan;30(1):70–82.
