# Supplementary material for: A reliable in vitro rumen culture system and workflow for screening anti-methanogenic compounds
Source: PLoS One. 2025 Dec 1;20(12):e0335844. doi: 10.1371/journal.pone.0335844 (PMC12668615; doi:10.1371/journal.pone.0335844)
Supplement: S1 Appendix — Consisting of S1 Fig., S2 Fig., S1 Table, S2 Table. (DOCX) [file pone.0335844.s007.docx]

Supplements:


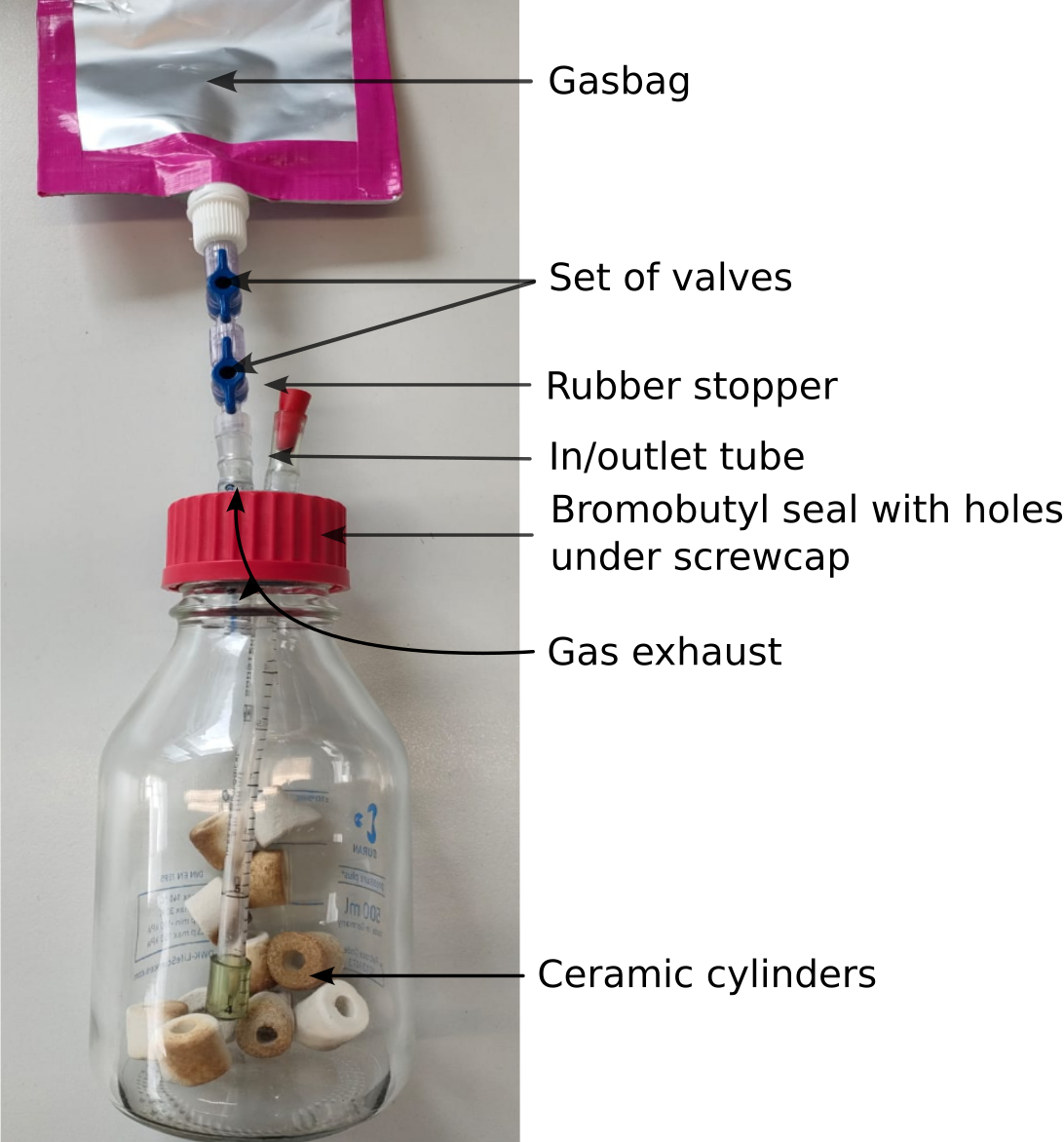
S1 Fig.: Prototype of the easily feedable rumen incubation flask. From top to bottom consisting of a reinforced 500 mL spout bag, luer-lock connectors and valves, two short PVC tubes mounted on serological pipettes, ceramic cylinders and the 500 mL glass bottle with bromobutyl seal and screw cap.

| 1H NMR | | | |
| --- | --- | --- | --- |
| 500 MHz, CDCl3 | δ 4.61 (t, J = 6.3 Hz, 1H) | 3.78 (t, J = 6.0 Hz, 1H) | 2.02 – 1.93 (m, 1H) |
| 13C NMR | | | |
| 126 MHz, CDCl3 | δ 70.4 | δ 58.9 | δ 29.8 |
| HRMS (electron ionisation) | | | |
| 121.9547 g/mol | | | |
| IR-Spectrometry | | | |
| 3331 (br w), 2966 (w), 2893 (w), 1618 (s), 1276 (s), 1046 (m), 856 (s), 759 (m) cm^-1^ | | | |

S1 Table: Analysis of 3-nitrooxypropanol by 1H NMR, 13C NMR, HRMS and IR spectrometry

Evaluation of the synthesised 3-NOP by NMR, HRMS and IR spectrometry shows low acetonitril impurities at δ 2.09 and of water at δ 1.59.

| Firmicutes |
| --- |
| Bacteroidetes |
| Spirochaeta |
| Verrucomicrobia |
| Patescibacteria |
| Fibrobacteres |
| Proteobacteria |
| Thermoplasmatota |
| Actinobacteriota |
| Elusimicrobiota |
| Chloroflexi |
| Bdellovibrionota |
| Cyanobacteria |
| Planctomycetota |
| Halobacterota |
| Desulfobacterota |
| Synergistota |
| Campilobacterota |
| WPS-2 |
| Unclassified |
| Methanobacteria |

S2 Table: List of reported taxa with 16S rDNA sequencing

S2 Fig.: Relative abundance of microbial taxa as determined by 16S rRNA amplicon sequencing over a course of 11 days. Error bars represent ± SEM (n=4) (A) Relative abundance of microbial taxa at the phylum level. (B) Relative abundance within the Methanogens. the Euryarchaeota phylum. BF: bromoform, IF: iodoform, 3-NOP: 3-nitrooxypropanol, BFOIL: bromoform in Oil.
