# Supplementary material for: A reliable in vitro rumen culture system and workflow for screening anti-methanogenic compounds
Source: PLoS One. 2025 Dec 1;20(12):e0335844. doi: 10.1371/journal.pone.0335844 (PMC12668615; doi:10.1371/journal.pone.0335844)
Supplement: Attachment — Submitted filename: PONE-D-25-30716.pdf [file pone.0335844.s008.pdf]

The authors tried to present an overview of anti-methanogenic compounds to mitigate the emission of these GHGs. However, there are several parts that need to be revised before publication.

1. The abstract of the article needs to be revised as it lacks key findings in the article.
2. The author should clarify why they chose T powder, BF, IF, 3-NOP, and rapeseed oil (NCOIL), and explain the rationale behind the concentrations of these supplements.
3. The image quality in the paper is poor. The author should revise these figures to improve readability.
4. The author should mention the material along with the method.
5. There is a lot of referencing to supplementary files (for example, S1 Fig, S2 File), but the main text lacks context.
6. There are various typo errors, such as "abbundances" (line 325). The author must improve and avoid these errors.
