# Supplementary material for: A reliable in vitro rumen culture system and workflow for screening anti-methanogenic compounds
Source: PLoS One. 2025 Dec 1;20(12):e0335844. doi: 10.1371/journal.pone.0335844 (PMC12668615; doi:10.1371/journal.pone.0335844)
Supplement: Attachment — Submitted filename: Response to Reviewers.docx [file pone.0335844.s010.docx]

Dear Dr. Abdullah,

Thank you for the opportunity to revise our manuscript. We are grateful to you and both reviewers for the thorough, constructive feedback. We have revised the paper extensively, and respond point-by-point below.

1. **PLOS ONE style**
   We reformatted the manuscript, figures, file names, and Supporting Information to meet PLOS ONE style. Figure references, numbering, abbreviations, units, and reference style were standardized.
2. **Role of funders**
   We confirm and have added the required statement:
   “**The funders had no role in study design, data collection, and analysis, decision to publish, or preparation of the manuscript.**”
   This text is included in the cover letter, per your instruction and in the submission form.
3. **Competing interests (patent)**
   We added a Competing Interests statement disclosing the patent related to the topic into the cover letter:
   “**We have read the journal's policy, and the authors of this manuscript have the following competing interests: The authors PL and BS are inventors on patent [AJ2811 PCT, Methods and compositions for reducing gas emission, and/or methane emission, and/or improving feed utilization of ruminants, European Patent Office, The Hague] related to the reduction of methane emission in ruminants. This does not alter our adherence to PLOS ONE policies on sharing data and materials.**”
4. **Figure copyright / CC BY 4.0**
   To eliminate any doubt regarding copyright, we recreated all elements of **Figure 2** using either (i) original artwork generated by the authors with ChatGPT-5.0 (licensed CC BY 4.0 [mentioned in Acknowledgements]) or (ii) vector graphics explicitly licensed under CC0/public domain. For example, the cow silhouette was taken from [PublicDomainVectors.org](https://publicdomainvectors.org/en/free-clipart/Ayrshire-cow/48144.html)
   Vector image of the cow: https://publicdomainvectors.org/en/free-clipart/Ayrshire-cow/48144.html
5. **Supporting Information captions**
   Captions for **S1–S6 Files** have been added at the end of the manuscript in accordance with PLOS ONE guidelines; in-text citations were updated to match.
6. **Protocols.io DOI and PDFs**
   All step-by-step protocols are attached as **S1–S6 Files (protocols.io PDFs)**, and cited in **Materials & Methods as a revision link**:
   The protocol described in this peer-reviewed article is published on protocols.io (<https://dx.doi.org/10.17504/protocols.io.kxygxwrnkv8j/v1>

# Response to Reviewers

We would like to sincerely thank both reviewers for their thoughtful and constructive comments. We greatly value the time and expertise you invested in evaluating our work. Your feedback has been valuable in enhancing the clarity, rigor, and overall quality of our manuscript.

We respond below in the order presented by the editor.

## Reviewer #1

1. The abstract was revised to include key quantitative results, specifically the maximum CH₄ reductions for each additive, with supporting statistical values.
2. The Results and Discussion sections were expanded to include detailed cross-system comparisons, effect sizes, and standard errors where available.
3. This phrase was replaced with a more precise description of the equipment used.
4. All formatting, subscripts, figure numbers, units, and taxonomy were standardized.
5. All figures were re-exported at higher resolution and checked with PACE for compliance, with improved clarity, and consistent formatting. Figure 2 was redrawn using CC BY 4.0–compliant artwork.
6. References were checked throughout and corrected for consistency and accuracy.
7. A comprehensive list of abbreviations was added before the references
8. The Discussion section was substantially restructured and expanded. More paragraphs were added for clarity, targeted reasoning was included, and statistical comparisons and microbiome interpretation were strengthened. Inoculum variability was justified, and a detailed limitations section was incorporated.

## **Reviewer #2**

1. The abstract was revised to include key results, including maximum CH₄ reductions.
2. A rationale paragraph was added to the Discussion, explaining the choice of compounds (AT, BF, IF, 3-NOP, NCOIL) and the basis for the selected concentration ranges.
3. Figures were replaced with higher-resolution versions and checked with PACE for compliance. Fig 2 was redrawn using CC BY 4.0–compliant artwork.
4. Materials are detailed in the deposited protocols on protocols.io, which are referenced in the Materials & Methods section and attached as S1–S6 Files with captions.
5. In the main manuscript there are no references to appended files or figures beyond the supporting information. As this is a protocol paper, the main text is not designed to be all-encompassing; the detailed step-by-step procedures are provided on protocols.io and as S1–S6 Files in line with PLOS ONE requirements.
6. The manuscript was thoroughly revised for language and formatting, with all typographical and taxonomic errors corrected.

We hope these revisions fully address the journal’s requirements. We look forward to your assessment.

Sincerely, Philip Laric, on behalf of all authors
